# The *AbDesign* computational pipeline for modular backbone assembly and design of binders and enzymes

**DOI:** 10.1002/pro.3970

**Published:** 2020-10-28

**Authors:** Rosalie Lipsh‐Sokolik, Dina Listov, Sarel J. Fleishman

**Affiliations:** ^1^ Department of Biomolecular Sciences Weizmann Institute of Science Rehovot Israel

**Keywords:** *AbDesign*, computational protein design, Rosetta, structural diversity

## Abstract

The functional sites of many protein families are dominated by diverse backbone regions that lack secondary structure (loops) but fold stably into their functionally competent state. Nevertheless, the design of structured loop regions from scratch, especially in functional sites, has met with great difficulty. We therefore developed an approach, called *AbDesign*, to exploit the natural modularity of many protein families and computationally assemble a large number of new backbones by combining naturally occurring modular fragments. This strategy yielded large, atomically accurate, and highly efficient proteins, including antibodies and enzymes exhibiting dozens of mutations from any natural protein. The combinatorial backbone‐conformation space that can be accessed by *AbDesign* even for a modestly sized family of homologs may exceed the diversity in the entire PDB, providing the sub‐Ångstrom level of control over the positioning of active‐site groups that is necessary for obtaining highly active proteins. This manuscript describes how to implement the pipeline using code that is freely available at https://github.com/Fleishman‐Lab/AbDesign_for_enzymes.

## 1 INTRODUCTION

Applied protein design methodology has made remarkable progress over the past few years.^1^ New design algorithms can now be generally applied to diverse proteins through automated web servers to improve protein stability,^2^, ^3^ affinity, specificity,^4^, ^5^ and catalytic efficiency.^6^ These approaches start from an existing (typically natural) protein structure and modify the amino acid sequence while minimally perturbing the backbone structure. Nevertheless, significant changes in protein activity or the design of completely new activities demand changes to the protein backbone to accurately position active‐site groups or encode large changes in substrate specificity. The design of new backbones, however, is vastly more complicated than fixed backbone design due to the many relevant degrees of freedom, especially in protein segments that lack secondary structure (loop regions).^7^ Additionally, particular care must be taken with protein active or binding sites which are often dominated by long loops. In these regions, mutations need to strike a fine balance between two possibly antagonistic features: conformational stability and molecular activity.^7^, ^8^, ^9^ Due to these complications, and though de novo protein design has shown huge progress over the past decade,^10^ the atomically accurate design of completely new backbones has been restricted to small proteins rich in secondary structures and exhibiting only short loops (typically ≤5 amino acids).

**FIGURE 1:**
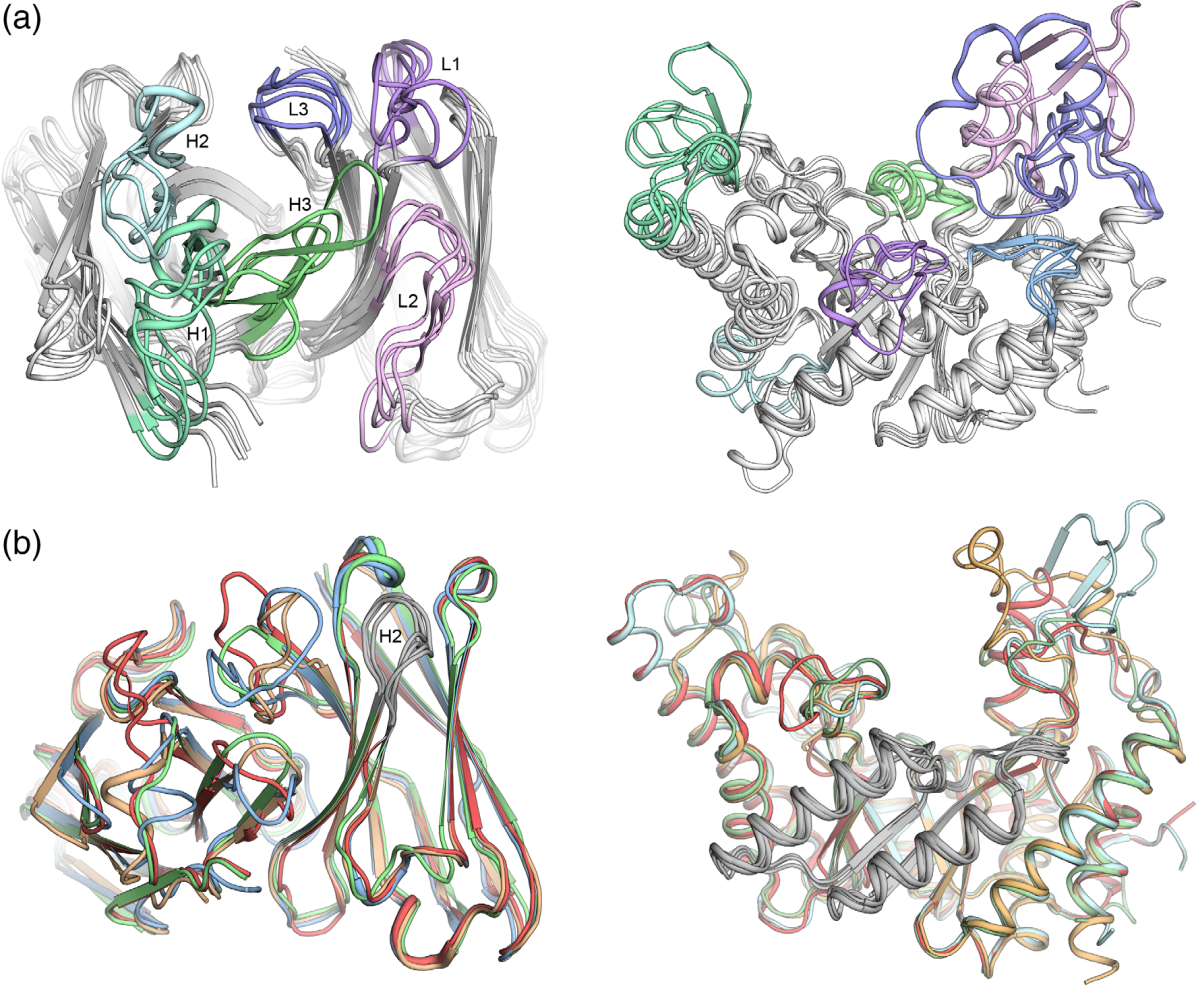
(a) The functional sites of many protein families are dominated by loop regions. Proteins from the same family have a similar overall fold, but the backbone diversity in their active sites provides a way to encode very large differences in substrate specificity or activity. Loops connecting aligned secondary structures are colored similarly. (*left*) The antibody variable domain with labeled CDRs; (*right*) TIM‐barrel GH10 xylanases. (b) Homologous proteins may comprise similar sub‐structures. Diverse protein backbones can have similar sub‐structures (colored in gray). (*left*) Four different antibodies, all having similar CDR H2 backbone (cluster H2‐10‐2 according to ref 15). (*right*) Four GH10 xylanase structures (PDB entries: 1R86, 2FGL, 1N82, and 1UQY) all have distinct structures but almost identical backbones in β–α Segments 5 and 6 (gray)

Given the difficulties of designing backbones from scratch, particularly in a protein active site, an alternative approach is to exploit the modularity and structural diversity of certain natural protein folds.^11^, ^12^, ^13^, ^14^ Antibodies of the immune system provide the quintessential example of a diverse and modular protein fold and have yielded very general lessons for backbone design. The antibody hypervariable and structurally diverse ligand‐binding surface comprises six regions (complementarity‐determining regions; CDRs L1‐3 and H1‐3; Figure 1a). Despite the length, fold‐complexity and hypervariability of the CDRs, their conformations are stable; this stability is largely encoded in evolutionarily conserved, long‐range interactions with the framework. Strikingly, five of the six CDRs (all but H3) can each be grouped into only a handful of so‐called canonical conformations.^16^ Thus, much of the structural diversity in antibodies is generated from the combination of only a small number of substructures, and the structural design principles that encode these substructures' conformations may be inferred from a structure‐bioinformatics analysis.^16^

Inspired by this natural approach to generate backbone diversity through the assembly of modular parts, we developed *AbDesign*,^17^, ^18^ an algorithm for combinatorial backbone assembly and design which relies on structure‐bioinformatics and atomistic design calculations. The algorithm starts by aligning homologous proteins (such as the antibody variable domain) and segmenting them according to points of maximum structure conservation (in antibodies, these may be the disulfide‐linked cysteines in the variable domain's framework). These segments are then computationally assembled to generate a huge diversity of new backbones, followed by Rosetta sequence design^19^ to optimize the stability and compatibility between the segments. Instead of allowing sequence optimization to search over all 20 amino acid identities at each position, however, *AbDesign* uses Position‐Specific Scoring Matrices (PSSMs) that are computed from a sequence alignment of structural homologs. Mutations that are rarely observed in the homologs are eliminated from the design options, thus guiding the atomistic design calculations towards implementing potentially stabilizing long‐range interactions, such as those between the CDRs and the framework. Furthermore, by eliminating rarely observed mutations, *AbDesign* reduces the chances of protein misfolding which has been a major obstacle in protein engineering and design.^20^, ^21^ The *AbDesign* approach thus generates a potentially very large number of backbones through the combination of naturally occurring ones followed by sequence design calculations to stabilize the entire protein including its active site. By controlling the fine details of the active‐site backbone, this approach provides the accuracy that is essential for encoding precise molecular recognition of substrates.

To recapitulate, there are two main methodological advances in *AbDesign* relative to fixed‐backbone design strategies: (a) the combinatorial expansion of backbone‐conformation space. Consider a family of homologous proteins comprising 20 nonredundant structures that can be segmented into four modular parts. Through modular assembly, we can obtain 20^4^ = 160,000 unique backbone conformations, exceeding the number of nonredundant structures in the PDB; and (b) optimizing the sequence using a constrained space of amino acids commonly observed in homologs. The resultant sequence subspace is likely to fold stably into the target conformation. *AbDesign* produced new atomically accurate antibodies.^18^ Furthermore, it yielded the first examples of designed ultrahigh specificity binding pairs comprising new backbones and accurate polar interaction networks at the protein‐interaction surfaces.^4^

*AbDesign* can be extended,^22^ in principle, to any modular protein family with structurally conserved sites. We verified *AbDesign's* generality by using it to automatically design new backbones for two unrelated TIM‐barrel fold family enzymes: glycoside hydrolase 10 (GH10) xylanases and phosphotriesterase‐like lactonases (PLL). Some designs exhibited strikingly different substrate specificities and high activity levels comparable to natural enzymes while differing from any known protein by more than 100 mutations, insertions and deletions. Remarkably, these enzymes were thermally stable and highly active without requiring iterative experimental mutagenesis and screening as has been the norm for protein‐design methodology.^23^ Despite the very large number of mutations from any known enzyme, the atomic accuracy of the design process was confirmed by crystallographic analysis. *AbDesign* thus opens the way to the application of protein design methodology to outstanding challenges in the design of protein function where sub‐Ångstrom accuracy is often a requirement. In this Tools contribution, we outline the steps that are required to apply *AbDesign* to a target enzyme family, noting that the same steps can be used, with necessary changes, to design binders and other proteins.

## 2 RESULTS

**FIGURE 2:**
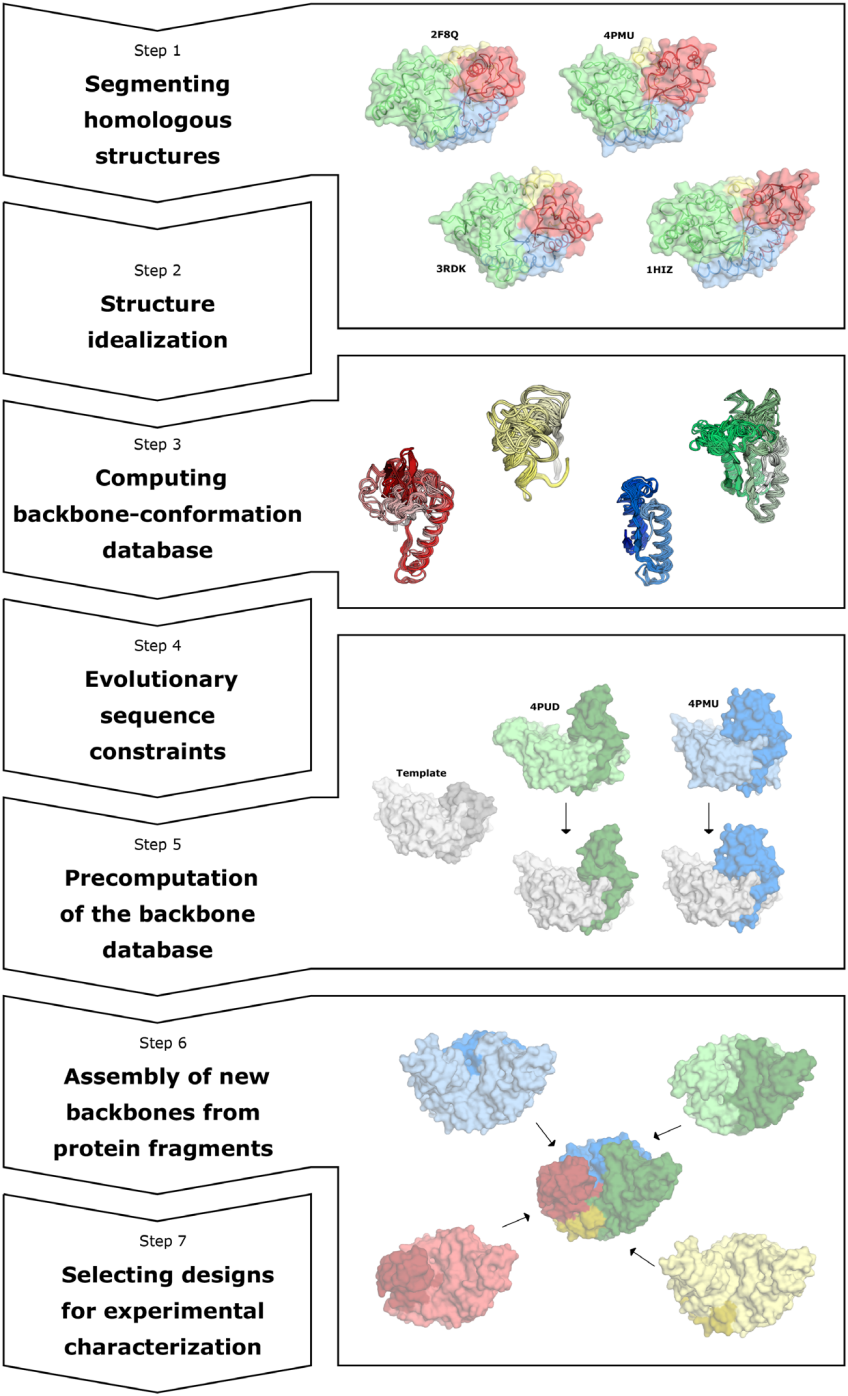
Key steps in modular assembly and design. Step 1: Homologous structures (labeled with their PDB entries) are colored by the segmentation scheme: Segments 1, 2–4, 5–6, and 7–8 are colored yellow, green, blue, and red, respectively. Step 3: Aligned fragments that belong to each of the segments. Coloring as in Step 1. Step 5: Each fragment is modeled in the context of the template structure. Step 6: Fragments from different homologs are assembled into a new, continuous backbone, and optimized by sequence design

**FIGURE 3:**
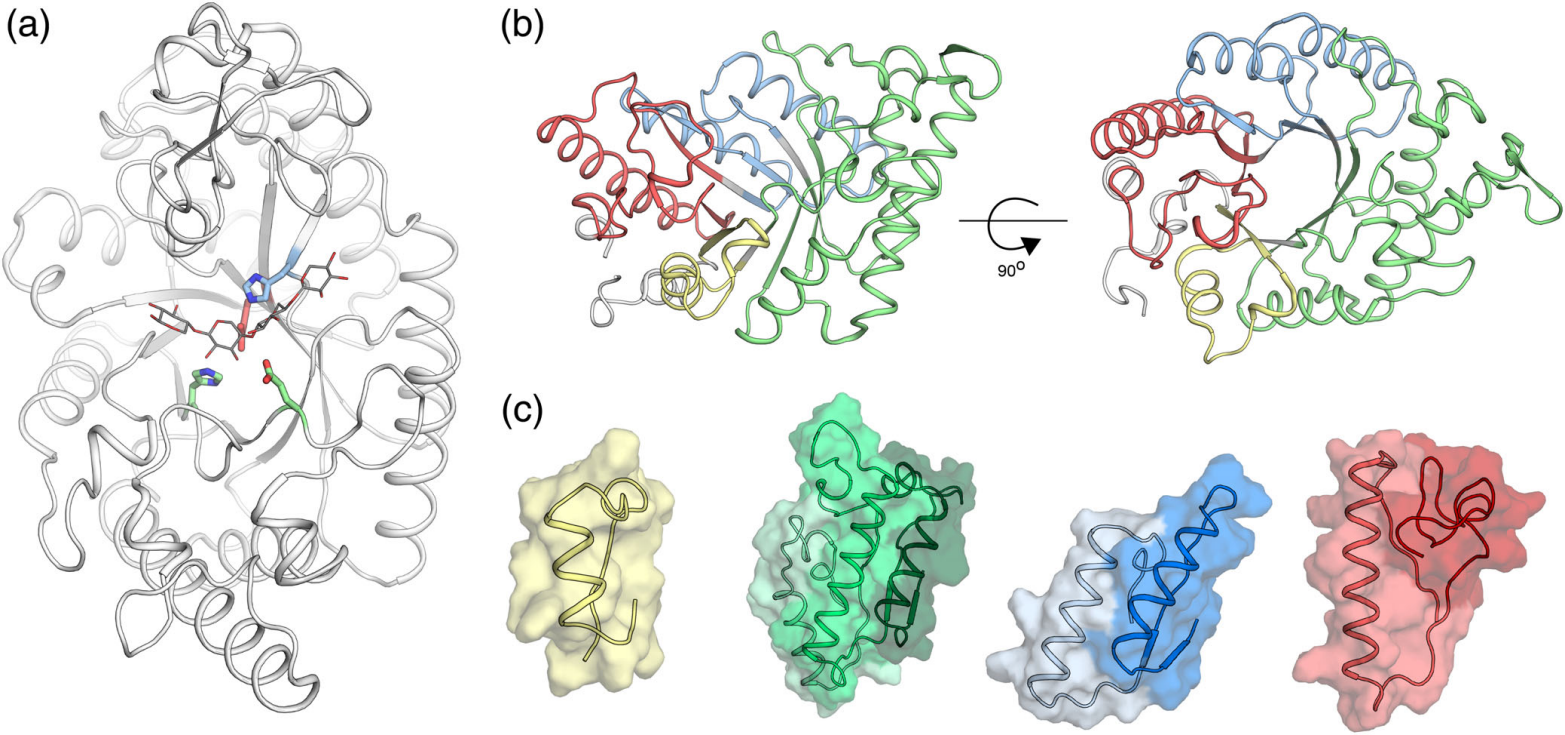
Structural segmentation of GH10 xylanases. (a) During all design steps, the conformations of the catalytic residues are held fixed to maintain the catalytic mechanism. Depicted is the template's structure (PDB entry: 3w24). The substrate is shown in thin white lines, and catalytic residues are colored according to the segmentation scheme in (b). (b) The template structure is colored according to the segmentation scheme: Segments 1, 2–4, 5–6, and 7–8 are colored yellow, green, blue and red, respectively. (c) β–α segments that form close‐packing interactions with one another are shown in different color tones based on the color‐scheme of (b)

In the sections below, we describe the steps needed to design a repertoire of enzymes exhibiting a diversity of backbones using the TIM‐barrel fold glycoside hydrolase 10 (GH10) xylanase family as a representative example (Figure 2 provides a visual aid that highlights the key steps in the pipeline). GH10 xylanases are monomeric enzymes that hydrolyze xylan via a retaining mechanism involving two Glu residues, one of which acts as a nucleophile and the other as a general acid/base.^24^ In all calculations, the key catalytic residues are held fixed in the sidechain conformations observed in a high‐resolution experimental structure (in this case, the two Glu and two additional supporting amino acids; Figure 3a). We note that active‐site positions may be designed if needed using other methods such as FuncLib^6^ or HotSpot Wizard.^25^

### 2.1 *Segmenting homologous structures*

We select a protein family of interest and download all available structures from freely available resources, such as the Pfam database of protein families.^26^ For the GH10 xylanase case, we downloaded all 143 available structures. Next, we manually select amino acid positions of maximal structure conservation based on structural alignment to serve as segmentation points. In antibodies, the disulfide‐linked cysteines of the variable domain serve as highly conserved segmentation points.^17^ Other folds, such as TIM‐barrels and β propellers, are much less conserved than antibodies and for each homologous family, the segmentation should be carefully considered; in some cases, several different segmentation schemes may need to be implemented as shown in References 4, 18, 22. It is also possible to segment protein structures into modular parts through energy‐based calculations that highlight densely packed regions.^27^ We have found that in TIM‐barrel families, the β‐strands are easily alignable since structural diversity is mainly observed in loop regions that connect the inner β‐strands and the outer α‐helices (these loop regions are critical determinants of substrate specificity; Figure 1a). While it is possible to segment a TIM‐barrel into eight segments, many segments form elaborate packing and polar interactions with spatially neighboring ones that may be difficult to accommodate if these segments were further cut. In GH10 xylanases, for example, β–α Segments 2, 3, 4 are closely packed as are 5, 6 and 7, 8 (Figure 3b,c). Thus, a structurally reasonable segmentation encompasses four segments: 1, 2–4, 5–6, and 7–8.

### 2.2 *Structure idealization*

All of the design calculations in *AbDesign* are carried out in torsion space only; thus, bond lengths and angles are not optimized during any of the calculations. Experimentally determined structures often exhibit deviations from ideal bond lengths and angles, and while some such deviations may be important structurally or functionally, these deviations are often due to the uncertainty of structure determination from low‐to‐medium resolution X‐ray crystallographic data. To reduce the noise in energy evaluations due to such deviations from ideal bond lengths and angles, we start by subjecting all of the structures to the Rosetta idealize procedure,^28^ which forces ideal bonds and relaxes the dihedral angles with harmonic backbone coordinate constraints to match the coordinates of the PDB entry. We have seen previously that in antibody structure prediction, idealization leads to model structures with excellent stereochemical properties^29^ as assessed by MolProbity.^30^ Ongoing research in our lab demonstrates that idealized protein structures can be used to design functioning enzymes (unpublished).

### 2.3 *Computing backbone‐conformation databases*

Next, we generate backbone‐conformation databases for each of the segments by segmenting the original structure files (PDB format) into fragments at the segmentation points. One of the structures of the protein family is arbitrarily designated to be the template (PDB entry 3w24 for GH10s)—the one according to which all other structures will be segmented. For that, we first align all structures to the template; then we extract from each structure all of the fragments.

To further refine the backbone database and promote accurate backbone assembly, a custom PyMOL script aligns all fragments belonging to the same segment such that the ends of the fragments are positioned identically, improving the seamless assembly of fragments into full‐length backbones. We note that for protein families with many representatives in the PDB, it may be beneficial to cluster the fragments and select a single representative for each cluster, thus reducing computational cost without compromising the backbone diversity (Figure 1b). For the GH10 xylanases, each segment has from 20 to 26 different clusters, and the sequences vary in length by up to 60 amino acids.

### 2.4 *Evolutionary sequence constraints*

The *AbDesign* approach uses position‐specific sequence constraints (based on PSSMs) for each backbone fragment based on the sequences observed in structurally homologous fragments. PSSM generation starts by assembling a multiple sequence alignment (MSA) based on the homologs. Sequences are typically extracted from the nr sequence database^31^ using BLAST at cutoffs of 35% sequence identity and ≥75% coverage of the template protein. In the GH10 case, this resulted in thousands of sequences of which we used the top 3,000.

### 2.5 *Precomputation of the backbone database*

Relaxing large backbone fragments, such as the ones used in *AbDesign*, is computationally very demanding. Modular assembly enables precomputing a relaxed conformation for each fragment, saving these conformations in a database, and quickly combining them during backbone assembly. The precomputation step enables the sequence design and ranking of hundreds of thousands of unique backbones. During precomputation, each segment of the template protein is removed one at a time, and each of the structurally aligned fragments from each homolog is relaxed using cyclic‐coordinate descent (CCD) backbone relaxation^32^ in the context of the template. In CCD, the protein mainchain is cut at a random position within the modeled fragment such that any change in the backbone dihedral angles is confined to that fragment only without impacting the remainder of the structure. Then, the entire fragment is minimized with constraints that reflect the positions and dihedral angles observed in the source structure and constraints that favor the seamless closing of the previously introduced cut. The protein's amino and carboxy termini are constrained at only one position each and are therefore more flexible than internal segments. *AbDesign* allows precomputing these tails with only one constraint. To facilitate refinement, we enable sequence optimization (constrained by the PSSMs) in the modeled fragment and its spatial vicinity. At the end of relaxation, we automatically verify that the backbone mainchain is continuous and that the root‐mean‐square deviation (rmsd) to the segment as observed in the source structure is low (<0.2 Å) before entering the segment's backbone torsion angles in the database.

### 2.6 *Assembly of new backbones from protein fragments*

The design process starts with the template structure, then replaces the different segments with fragments from the precomputed fragment databases. Whenever a fragment is replaced, *AbDesign* automatically changes the PSSM constraints to reflect the newly assembled structure. Then, the new fragment and its spatial vicinity are sequence‐optimized for the backbone structure subject to these PSSMs. For GH10 xylanases, 3,000 backbones were generated, but in cases where the number of fragments in each segment is modest, it may be best to enumerate all possible backbones to ensure that the most optimal ones are selected for further analysis. When backbone design is complete and all segments are replaced with fragments from the database, we use PROSS stability design^2^, ^20^ to increase the stability of the new backbone and improve the fit between the different fragments to generate a seamless structure. By default, PROSS produces seven designs^2^ and we typically choose the second‐to‐highest design in terms of the number of mutations. We have observed that the PROSS‐design step, which may result in dozens of new mutations, is essential for high stability and protein expressibility.^22^

### 2.7 *Selecting designs for experimental characterization*

The backbone design algorithm described above may generate hundreds of thousands of unique designs, and a subset is chosen for experimental characterization according to the experimental screening capacity. In the designed enzyme set described in Reference 22, we ranked the designs based solely on their system energy. We note, however, that while the Rosetta energies estimate protein stability, they do not reflect functional constraints. For example, to exhibit high enzymatic activity, the catalytic pocket should be preorganized and all catalytic residues must adopt the desired conformation at sub‐Ångstrom accuracy. Thus, designs with inaccurate active‐site constellations may be eliminated. Other important features may include the internal packing density of the protein (for instance, using the Rosetta packstat measure^33^), shape complementarity with a binding partner,^34^ correct docking of a small molecule to its binding pocket, the active‐site residues' preorganization^35^ and any other feature relevant for the designed system.

When applying several criteria in design selection, strict cutoffs are likely to drastically decrease the pool of designs. Instead, one may combine the different criteria using a “fuzzy‐logic” expression.^36^ Briefly, we can define the design's fitness relative to an arbitrary criterion through a logistic function as:

$$f=\frac{1}{1+e^{\frac{x-\mu }{\sigma }}}$$

where *x* is the value of the criterion (for instance, the design's computed binding energy to the ligand) and *μ* and *σ* are the mean and standard deviation of the criterion in the design ensemble, respectively. The fitness, *f*, asymptotically approaches 1 at negative *x* values and 0 at positive *x* values. Undesirable features, such as binding energy to off‐target substrates, may be encoded by simply negating *x*. We can further combine several such fitness scores, each expressing a different feature, into an optimization objective function by multiplying the individual fitness terms:

$O\left(\text{design}\right)=\prod_{x\in \text{features}}f\left(x\right)$

Designs with objective‐function scores close to 1 find a favorable compromise among all of the features, whereas designs with scores close to 0 exhibit at least one poor feature. Examples for using fuzzy‐logic design are discussed in References 17, 18, 36.

## 3 DISCUSSION

Until recently, the control that computational design methods exercised over the protein backbone degrees of freedom was quite limited. The recent breakthroughs in the application of de novo protein design methodology to increasingly complex problems are very encouraging,^37^, ^38^, ^39^, ^40^, ^41^, ^42^ but these applications are still restricted to small proteins that are dominated by secondary structure elements. By contrast, proficient and versatile binders and enzymes are almost exclusively large and their active sites are often dominated by long (though highly structured) loop regions. The large size and fold‐complexity of many binders and enzymes likely reflect a physical requirement for a high density of functional groups at the active site. Dense and preorganized constellations of functional groups may demand loop regions that are structurally stabilized by a large and rigid scaffold.^11^, ^22^

The *AbDesign* algorithm leverages the fact that many functionally versatile protein folds are modular—likely because modularity facilitates the evolution of new activities^43^—to generate a vast space of new backbones. Although the focus of this manuscript was on combining fragments from homologous family members, we note that we have used *AbDesign* to recombine fragments from non‐homologous proteins to generate new backbones and polar interaction networks in binding pairs.^4^ Furthermore, recent proteome‐wide analyses have detected significant homology between protein fragments that come from evolutionarily very distant families.^44^, ^45^, ^46^, ^47^ These observations suggest that modular assembly can be used to generate structural innovations beyond the family and even fold level. Thus, *AbDesign* provides a practical route to designing large‐scale changes in natural enzymes and binding proteins.

Backbone design in catalytic pockets and binding surfaces paves the way to address long‐standing challenges in protein design. For instance, *de novo* enzyme design has so far resulted in enzymes that exhibited low efficiency and required further optimization by directed evolution.^48^, ^49^ Active‐site backbone design could provide the sub‐Ångstrom accuracy that is needed for the design of high‐efficiency enzymes and binders.

## 4 MATERIALS AND METHODS

All design calculations were performed using Rosetta, freely available for academic and non‐profit users. Scripts, files, and command lines to generate a backbone repertoire can be found at https://github.com/Fleishman-Lab/AbDesign_for_enzymes . Backbone clustering was performed using MaxCluster,^50^ with maximum linkage hierarchical clustering, sequence‐independent mode and an rmsd fit. PSSMs are generated as described in Reference 2 .

## AUTHOR CONTRIBUTIONS

**Rosalie Lipsh‐Sokolik:** Conceptualization; data curation; formal analysis; investigation; methodology; software; validation; visualization; writing‐original draft; writing‐review and editing. **Dina Listov:** Data curation; formal analysis; investigation; methodology; validation. **Sarel Fleishman:** Conceptualization; funding acquisition; investigation; methodology; project administration; supervision; validation; writing‐original draft; writing‐review and editing.
